# Disparities in Screening: Can Education Address Imbalance?

**DOI:** 10.1016/j.atssr.2024.07.001

**Published:** 2024-07-15

**Authors:** Justin A. Olivera, Rajika Jindani, Mara B. Antonoff

**Affiliations:** Albert Einstein College of Medicine, Bronx, New York; Department of Thoracic and Cardiovascular Surgery, University of Texas MD Anderson Cancer Center, 1400 Pressler St, Unit 1489, Houston, Texas 77030

To the Editor:

We commend Hutchings and colleagues^1^ for their insightful study in *The Annals of Thoracic Surgery Short Reports* on the effect of education on subsequent willingness to undergo lung cancer screening. Hutchings and colleagues^1^ have shown that although willingness may not be a driving factor in disparate screening rates among Black Americans, it could potentially influence sex-related disparities.

This study identified racial differences in perceived behavioral control regarding screening, with Black participants demonstrating greater perceived control, whereas other variables were comparable across racial groups. Additionally, the study identified more positive attitudes and intentions toward screening among women.

This report is relevant given the contemporary focus on race- and sex-related screening disparities. Hutchings and colleagues^1^ suggest a similar willingness for screening among Black and White respondents after education, thus generating the impetus to elucidate other factors driving the persistent disparities in screening rates. Recent work from Potter and colleagues^2^ proposes eligibility criteria as a likely source of such disparities. Women (regardless of race) and Black patients (regardless of sex) tend to have smoked significantly fewer pack-years at diagnosis, a finding implying that the 20-pack-year eligibility requirement carries bias that may drive disparities. Hutchings and colleagues^1^ recommend smoking duration as an equalizing risk factor. This approach holds considerable promise among patients who smoke, yet a need remains for novel paradigms addressing the rising number of nonsmoking women facing lung cancer at younger ages.^3^

We appreciate the candor of Hutchings and colleagues^1^ regarding the study’s limitations. The unbalanced representation of Black (n = 52) and White (n = 173) participants was attributed to recruitment difficulties, a consistent challenge in thoracic oncology research. Hutchings and colleagues^1^ suggest that targeted recruitment strategies could increase participation among underrepresented minorities. We also encourage exploring successful strategies from other specialties to identify additional opportunities, such as emphasizing “patient-perceived value” as a motivating factor for participation.^4^

This contribution from Hutchings and colleagues^1^ provides important context to the ongoing discourse on screening disparities. These findings underscore the importance of screening education and the need for interventions regarding screening disparities. We thank Hutchings and colleagues^1^ for their contribution and look forward to more work in this realm.
